# Behavioral biases and heuristics in perceptions of COVID‐19 risks and prevention decisions

**DOI:** 10.1111/risa.13882

**Published:** 2022-01-29

**Authors:** W. J. Wouter Botzen, Sem J. Duijndam, Peter J. Robinson, Pieter van Beukering

**Affiliations:** ^1^ Institute for Environmental Studies (IVM) Vrije Universiteit Amsterdam The Netherlands; ^2^ Utrecht University School of Economics (U.S.E.) Utrecht University Utrecht The Netherlands; ^3^ Risk Management and Decision Processes Center, The Wharton School University of Pennsylvania Philadelphia USA

**Keywords:** coronavirus, COVID‐19, decision making, heuristics, risk perceptions

## Abstract

This study adds to an emerging literature on the factors associated with individual perceptions of COVID‐19 risks and decision‐making processes related to prevention behaviors. We conducted a survey in the Netherlands (*N *= 3600) in June–July 2020 when the first peak of COVID‐19 infections, hospitalizations, and deaths had passed, and lockdown measures had been eased. Dutch policies relied heavily on individual prevention behaviors to mitigate a second infection wave. We examine whether biases and heuristics that have been observed in how people perceive and respond to other risks also apply to the newly emergent risks posed by COVID‐19. The results indicate that people simplify risk using threshold models and that risk perceptions are related with personal experiences with COVID‐19 and experiences of close others, supporting the availability heuristic. We also observe that prevention behavior is more strongly associated with COVID‐19 risk perceptions and feelings toward the risk than with local indicators of COVID‐19 risks, and that prevention behavior is related with herding. Support for government lockdown measures is consistent with preferences that may contribute to the not‐in‐my‐term‐of‐office bias. In addition, we offer insights into the role of trust, worry, and demographic characteristics in shaping perceptions of COVID‐19 risks and how these factors relate with individual prevention behaviors and support for government prevention measures. We provide several lessons for the design of policies that limit COVID‐19 risks, including risk communication strategies and appeals to social norms. Perhaps more importantly, our analysis allows for learning lessons to mitigate the risks of future pandemics.

## 1 INTRODUCTION

The global impacts of COVID‐19 illustrate that pandemics are a major societal risk causing substantial consequences for human health and the economy (Tisdell, 2020). Population growth, globalization, and related international travel, transport, and tourism flows may all contribute to the risks of future pandemics (Barouki et al., 2021). Moreover, climate change may enhance the spreading of infectious diseases (Intergovernmental Panel on Climate Change, 2014). Government prevention measures, such as lockdowns, appeared to be an effective, but in the case of lockdowns expensive, way to limit COVID‐19 infections. Moreover, the COVID‐19 pandemic has underscored the importance of individual behavior in spreading the coronavirus, with regular human contact resulting in exponential growth of infections (Duffey & Zio, 2020). By contrast, individual prevention actions as well as support and compliance with lockdown measures have proven to be effective in flattening the curve of rising infections (Stavroglou Ayyub et al., 2021). These individual prevention actions are most effective if many people adopt them. These experiences illustrate the importance of understanding individual decision‐making processes about COVID‐19 prevention actions and factors that may influence these actions, such as risk perceptions. Such insights may contribute to the design of policies that limit COVID‐19 risks, but perhaps more importantly allow for learning lessons to mitigate the risk of future pandemics.

We implemented a survey in the Netherlands (*N *= 3600) in June–July 2020, during which lockdown measures had been eased and the first peak of COVID‐19 infections, hospitalizations, and deaths had passed. Strict lockdown measures were implemented during the first wave from the second half of March until the end of May. These measures included the closing of shops and restaurants, the prohibition of group gatherings, workplace restrictions, and eventually the mandatory wearing of mouth masks in public spaces. Most of these measures were (partly) relaxed at the time of the survey, but social distancing, limiting group sizes, and wearing masks in public transport were still required.

We may have observed higher concerns about COVID‐19 if we would have implemented the survey at the start of the pandemic. In the Netherlands, compliance with COVID‐19 prevention guidelines was high during the first phase of the pandemic that started in March when there was a high sense of urgency. A survey study in three countries—including the Netherlands—conducted in March 2020 by Meier et al. (2020) revealed widespread support for the government measures that were in place to limit COVID‐19 infections. This result is consistent with research in 10 countries that demonstrated high concern about COVID‐19 in March–April (Dryhurst et al., 2020). Moreover, Siegrist et al. (2021) found that Swiss people in this period had a high a concern for others becoming infected, and Zanin et al. (2020) reported high levels of uncertainty and fear regarding COVID‐19 among Italian households.

To prevent a second wave of COVID‐19 infections, individual prevention actions such as regular handwashing and social distancing, were particularly central to government policies in the Netherlands and various other countries (Meier et al., 2020). During this second phase, perceived health risks may have declined, however, in the absence of stringent lockdown measures individual prevention behaviors were the main means to prevent another wave of infections. Therefore, this moment presents an interesting setting for analyzing decision‐making processes that offer insights into why some individuals have high COVID‐19 risk perceptions and regularly take prevention actions, whereas others may have low risk perceptions and rarely engage in prevention measures. At the time we designed our survey, little was known about COVID‐19 risk perceptions as well as prevention behaviors and their drivers.

Extensive research in the fields of psychology and behavioral economics have examined how individuals perceive and act upon other risks than COVID‐19 (Kahneman, 2011; Slovic et al., 2004) such as natural disasters (Meyer & Kunreuther, 2017). This research may provide important lessons for how individuals perceive and respond to a relatively new risk, such as COVID‐19. Previous research has shown that individuals often do not perceive risks in the same way as experts do (Botzen et al., 2015; Slovic, 2000). Given the complexity of processing risks, individual risk perceptions and responses can be driven by intuitive thinking that is associated with simplifying decision heuristics and systematic behavioral biases (Kahneman, 2011; Kunreuther, 2018). Based on this literature, Botzen Duijndam et al. (2021) identified behavioral biases that are likely to influence individual perceptions of risks associated with COVID‐19 and decisions about taking actions to limit these risks. The same authors also drew parallels between these biases in the contexts of climate change and the COVID‐19 pandemic. The focus of our study is on the empirical testing of a variety of hypotheses about these heuristics and biases in individual perceptions of risks associated with COVID‐19 and related prevention actions. Although similar research has been done related to other types of risk, such as flood risk (see, e.g., Bubeck et al., 2012), the unique manifestation of COVID‐19 risk and the far‐reaching global impact of the COVID‐19 pandemic make it highly relevant to understand to what extent existing knowledge about risk perceptions and behavior pertain to COVID‐19 as well, and if there are particular differences that have to be taken into account in future research and policymaking.

The first studies in this direction show that intuitive thinking and heuristics may also influence the way that individuals perceive risks associated with COVID‐19 (Dryhurst et al., 2020; Siegrist et al., 2021; Wong et al., 2021). Findings by Wong et al. (2021) indicate how the heuristic processing of information on COVID‐19 by individuals in the United States is exacerbated by information that blames China for the pandemic, which is mediated by negative emotions and risk perceptions. Siegrist et al. (2021) underscore the importance of the affect heuristic in Switzerland, whereby negative emotions toward the risk determine the perceptions of the health consequences of COVID‐19; they also highlight the value of various indicators of trust in shaping individual risk judgments and prevention behavior. Dryhurst et al. (2020) find that personal experience with the coronavirus positively influences individual perceptions of COVID‐19 risk, which points toward the availability heuristic.

Our study provides a more comprehensive empirical assessment of the availability heuristic by examining the relationships of COVID‐19 risk perceptions with personally experiencing COVID‐19 infection, experienced illness, experienced costs as well as experiences by close others. In addition, we investigate the simplification of risk through the application of threshold models (Slovic et al., 1977). Moreover, we estimate how prevention behavior relates to herding, and social norms (Van Bavel et al., 2020) as well as political preferences that may contribute to the not‐in‐my‐term‐of‐office (NIMTOF) bias (Kunreuther & Useem, 2010). Apart from heuristics and biases, we also examine how individual prevention behavior relates to risk perceptions. In addition to our core hypotheses, we examine how risk perceptions and prevention behavior relate to local indicators of COVID‐19 risks—encompassing positive tests, hospitalizations and deaths from COVID‐19—and other variables capturing trust in the government, and sociodemographic characteristics, including individual risk attitudes.

We focus on a variety of dimensions of risk perceptions, which have generally been identified in the literature as important risk perception indicators (Kellens Terpstra et al., 2013). These dimensions include the perceived likelihood of becoming infected by COVID‐19 (Dryhurst et al., 2020), perceived consequences (Siegrist et al., 2021), as well as feelings such as worry toward the risk (Loewenstein et al., 2001). Furthermore, to obtain a comprehensive assessment of prevention, we elicited how often our respondents engage in a variety of individual COVID‐19 risk prevention actions as well as their support for government prevention through lockdown measures.

The remainder of this article is structured as follows. Section 2 presents the behavioral heuristics and biases and our related set of hypotheses. Section 3 includes a description of the survey and statistical methods. Section 4 is focused on the results for the various risk perception dimensions and prevention actions. In Section 5, these results are discussed in relation to our hypotheses and other studies. Section 6 concludes and gives recommendations for policymakers who aim to improve individual preparedness for pandemic risks such as COVID‐19.

## 2 BEHAVIORAL HEURISTICS AND BIASES AND HYPOTHESES

Based on decades of research in psychology and behavioral economics, Kahneman (2011) has identified two modes of thinking that explain how individuals perceive and make decisions about risk, which are System 1 and System 2. These modes have also been called the experiential and the analytical systems by Slovic et al. (2004). System 1 represents intuitive thinking processes and operates automatically and quickly with little or no effort and no sense of voluntary control. System 2 represents analytical risk assessments and allocates attention to the effortful mental activities that demand it, including simple or complex calculations or formal logic. System 1 thinking has been associated with heuristics that simplify complex decisions for example, assessing the COVID‐19 infection likelihood and consequences, into simple judgments or “rules‐of‐thumb” (Tversky & Kahneman, 1974). System 1 thinking is likely to be important for unfamiliar risks. The use of heuristics can cause systematic behavioral biases. Behavioral biases are the errors in judgment, such as errors in probabilistic reasoning, or risk perceptions that deviate from those that would lead to “optimal” behavior in the sense of expected utility theory (Gigerenzer, 1991).

A variety of studies reviewed heuristics and biases that are commonly associated with risk perceptions (Kunreuther, 2018; Meyer & Kunreuther, 2017). Our core hypotheses are structured around four behavioral biases and heuristics that have been shown to influence decision making toward various risks and have been identified by Botzen et al. (2021) as likely to influence COVID‐19 risk perceptions and prevention behavior. As described below with the supporting literature, these behavioral biases and heuristics include the availability heuristic, simplification of risk and use of threshold models, herding, and the NIMTOF bias. Apart from these biases and heuristics, we examine how individual prevention behavior and support for government prevention measures are related with risk perceptions.

### 2.1 Availability heuristic

The availability heuristic posits that an individual's perceived probability of an event occurring and its perceived consequences depend on the ease with which the event comes to mind, often through personal experience (Tversky & Kahneman, 1973). Biases resulting from people's reliance on the availability heuristic have been observed for other risks, for instance related to medicine and climate change. In medicine, the availability heuristic has been found to affect physicians’ reasoning when diagnosing a particular disease, with physicians being more likely to diagnose a particular disease after having had a recent experience with the disease in previous patient cases, leading to diagnosing errors (Mamede et al., 2020). For climate change, the perception of natural disaster risks such as floods is often much higher after a recent experience with the disaster (Botzen et al., 2015; Siegrist & Gutscher, 2006). The availability heuristic could similarly apply to risk perceptions related to COVID‐19, with personal experience increasing risk perceptions of the virus and its consequences (Dryhurst et al., 2020). This premise is tested in our study with the following hypotheses:

H1a: Personal experience with COVID‐19 infection is positively related with the perceived probability of COVID‐19 infection.
H1b: Knowing close others who experienced COVID‐19 infection is positively related with the perceived probability of COVID‐19 infection.
H1c: Personally experiencing severe illness from COVID‐19 is positively related with the perceived health consequences from COVID‐19 infection.
H1d: Knowing close others who experienced severe illness from COVID‐19 that resulted in their death is positively related with the perceived health consequences from COVID‐19 infection.
H1e: Personally experiencing financial costs from COVID‐19 is positively related with the perceived financial consequences from COVID‐19 infection.

### 2.2 Simplification of risk and use of threshold models

Individuals are likely to make choices without considering the full risk distribution of probabilities and consequences of an event occurring. Instead, many people rely on threshold models and refrain from risk‐reducing behavior when the probability of an event falls below the threshold level of concern. The simplification of risk by applying the threshold models heuristic implies that individuals neglect the potential consequences of a risk once they treat its probability as being below some threshold level of concern (Slovic et al., 1977). This simplification is often observed in risk behavior related to natural disasters, whereby people do not want to protect themselves against risk when the perceived probability of a disaster is very low (Kunreuther, 2020; Robinson & Botzen, 2019). A similar line of reasoning could hold for COVID‐19 when people perceive the probability of becoming infected by COVID‐19 as lower than their threshold level of concern. In that case, they will downplay the consequences of the virus and have lower demand for COVID‐19 protection measures. H2a and H2b examine this:

H2a: Treating the COVID‐19 infection probability as too low to be concerned about is negatively related with the perceived health and financial consequences of COVID‐19 infection.
H2b: Treating the COVID‐19 infection probability as too low to be concerned about is negatively related with the demand for individual and government COVID‐19 protection measures.

### 2.3 Risk perception and demand for prevention

Although the influence of risk perceptions on individual risk reduction behavior and support for government prevention measures is not necessarily a bias in itself, this relationship can imply that individual biases in risk perception also translate into prevention actions. Risk perception as a driver for private risk mitigation measures or support for risk mitigating policies has been extensively studied for risks such as climate change (van Valkengoed & Steg, 2019), flooding (Bubeck et al., 2012), and droughts (Khan et al., 2020). Similarly, in the context of COVID‐19, perceptions of COVID‐19 risks are observed to be positively related with individual prevention actions as well as support for government prevention measures (de Bruin & Bennett, 2020; Dryhurst et al., 2020; Siegrist et al., 2021). A positive relationship between risk perceptions and a preference for risk mitigation measures can be explained by the “motivational hypothesis.” This hypothesis states that people support or take mitigation measures to lower the particular risk that they perceive as being high (Weinstein et al., 1998). Risk perceptions, which are also denoted as threat appraisals, are a central component of psychological and economic theories of decision making under risk, such as Protection Motivation Theory (Rogers, 1975, 1983), the Health Belief Model (Becker, 1974), and Subjective Expected Utility Theory (Savage, 1954). Following these theories, we can expect that people with higher risk perceptions of COVID‐19 are more likely to take personal precautionary measures and to support government prevention measures, as stated in H3a and H3b.^1^

H3a: The perceived consequences and worry for COVID‐19 infections are positively related with the frequency that individuals apply measures that prevent COVID‐19 infection.
H3b: The perceived consequences and worry for COVID‐19 infections are positively related with support for government prevention measures.

### 2.4 Herding

The behavior of other people is often mirrored by individuals, which is especially the case when an issue is characterized by high uncertainty or risk. Such following of social norms instead of rational risk assessments is referred to as herding behavior (Kunreuther, 2018). For instance, people have been found to be more likely to implement flood adaptation measures (Lo, 2013) or install solar panels (Bollinger & Gillingham, 2012) when people close to them also undertake these measures. A similar argument is that people commit to behavior that prevents COVID‐19 transmission when such conduct is viewed as socially desirable behavior by others (Soofi et al., 2020), that is, there is an (injunctive) social norm to do so (Van Bavel et al., 2020). This premise is tested by the following hypothesis:

H4: The implementation of measures that prevent infection from COVID‐19 is positively related with believing that others think that this is the right thing to do.

### 2.5 Not‐in‐my‐term‐of‐office bias

In addition to individual prevention behaviors, government prevention measures play an important role in limiting COVID‐19 risks. Government decision making is likely to be influenced by the preferences of their voters. A complicating factor is that some risks such as pandemics generally have a low risk of occurring within a political term of office. For this reason, some politicians refrain from implementing the necessary expensive measures to prevent these risks from occurring, as politicians will likely not reap the benefits of these investments while in office. Instead, investing in measures that yield visible short‐term benefits for voters is often more appealing when considering reelection. This situation, which is characterized by underinvestment in risk prevention measures, has been coined the “NIMTOF” bias (Kunreuther & Useem, 2010). NIMTOF is visible in the case of climate change; despite the substantial future risks of climate change, governments are often still reluctant to implement necessary mitigation and adaptation measures. Although some governments implemented costly lockdown measures to limit COVID‐19 infections and fatalities during the pandemic, governments around the world were also generally ill‐prepared for the COVID‐19 pandemic, despite having been warned for decades by virologists about the risks of pandemics emerging in the future (Sands et al., 2016). COVID‐19 prevention measures are a means of preventing the future infection risks of the virus. Hence, we can expect that individuals who favor present‐biased politicians (i.e., those acting according to the NIMTOF bias) are more likely to oppose government‐imposed COVID‐19 prevention measures. H5 therefore states that:

H5: Preferences for present‐biased politicians are negatively related with support for government COVID‐19 prevention measures.

## 3 METHODOLOGY

### 3.1 Description of the survey method and participants

An online survey was conducted among a sample of 3600 Dutch homeowners who were recruited through random draws of the survey panel of Panel Inzicht in June–July 2020 (https://panelinzicht.nl).^2^ This method of conducting a survey online allowed for obtaining a large and diverse sample at a relatively low cost and prevented interviewer effects (Horton et al., 2011). The survey was conducted after the first peak of COVID‐19 infections in the Netherlands had passed. The first wave of COVID‐19 infections and deaths in the Netherlands peaked with 1394 infections on April 10 and with 178 deaths on April 2. On the day of the start of the survey, June 18, only 83 new infections and five deaths were reported (RIVM, 2021a).

Following some brief introductory text that guaranteed the anonymity of respondents and stated that the data would be strictly used for scientific purposes only, socioeconomic questions were presented, and location data were obtained according to the respondents’ postal codes. Note that the analysis in the main results sections of this article is based on 2705 observations because of some missing values with regard to this location data and incomes. According to the data upon which we base our analysis, 44% are female, the average age is 50 years, 43% completed higher education (obtained either a bachelor's, master's, or PhD degree), and the median selected after tax monthly household income category is between €3000 and €3999.^3^ In the actual Dutch population, the average age is 42, 30% completed higher education, and the median income is about €2200. Hence, our sample overrepresents higher income and education levels as well as older individuals, which may be due to the fact that we targeted homeowners. Nevertheless, our sample represents a large share of the Dutch population, as 57% of households own their own house in the Netherlands.^4^

The original survey questions are given in Appendix A in the Supporting Information. Table 1 defines how the variables are derived from the survey questions and coded. They are subsequently explained for the variables used for testing our hypotheses (Section 3.2) and other explanatory variables (Section 3.3).

**TABLE 1:** Variable definitions and coding

Variable	Coding
Perceived COVID‐19 infection probability (qualitative)	How likely do you think it is that you will personally be infected by the coronavirus? 1 = very unlikely, to 5 = very likely.
Perceived COVID‐19 infection probability (quantitative)	Within the next year, what is your best estimate of the likelihood that you will personally be infected by the coronavirus? 1 = Less than 1 in 100,000, to 6 = greater than 1 in 10.
Perceived health consequences COVID‐19	Suppose you would be infected by the coronavirus, how sick do you expect to get from the virus? 1 = not sick at all, to 5 = extremely sick.
Perceived financial consequences COVID‐19	Suppose you would be infected by the coronavirus, what financial consequences do you expect for you personally from this infection, for example due to medical costs or income loss? 1 = no financial costs, to 5 = very high financial costs.
Handwashing	How often do you take the following actions to prevent becoming infected by the corona virus? I follow official guidelines to regularly wash hands for at least 20 seconds. 1‐never, to 5 = always.
Stay home	How often do you take the following actions to prevent becoming infected by the corona virus? I stay inside my house as much as possible. 1‐never, to 5 = always.
No guests	How often do you take the following actions to prevent becoming infected by the corona virus? I refrain from receiving guests in my home. 1‐never, to 5 = always.
Social distancing	How often do you take the following actions to prevent becoming infected by the corona virus? I follow official guidelines to keep distance from other people when I go outside. 1‐never, to 5 = always.
Avoid public transport	How often do you take the following actions to prevent becoming infected by the corona virus? I do not use public transport because of the coronavirus. 1‐never, to 5 = always.
Local positive test rate	Cumulative number of COVID‐19 infections per citizen at the date of the survey, relative to the Dutch average. Data collected at municipality level.
Local death rate	Cumulative number of COVID‐19 deaths per citizen at the date of the survey, relative to the Dutch average. Data collected at municipality level.
Local hospitalization rate	Cumulative number of COVID‐19 hospitalizations per citizen at the date of the survey, relative to the Dutch average. Data collected at municipality level.
Personally experienced infection	Have you personally been infected by the corona virus? 1 = no, 2 = uncertain, 3 = thinks so, 4 = convinced, 5 = confirmed.
Others experienced infection	Has at least one of your household members, close relatives or close friends been infected by the corona virus? 1 = no, 2 = uncertain, 3 = thinks so, 4 = convinced, 5 = confirmed.
Personally experienced sickness	How sick did you get from the (possible) infection with the coronavirus? 1 = was not infected, 2 = not sick after infection, 3 = a little bit sick, 4 = quite sick, 5 = very sick, 6 = extremely sick.
Experienced the death of someone close	1 = a household member, close relative or close friend died as a consequence of the coronavirus, 0 = otherwise.
Personally experienced costs from COVID‐19:	Did your household incur costs as a consequence of the coronavirus for example because of the loss of employment, temporary leave, or medical expenses? (no = excluded baseline).
Medical costs	1 = yes, because of medical expenses, 0 = otherwise.
Costs from temporary leave	1 = yes, because of temporary leave, 0 = otherwise.
Costs from employment loss	1 = yes, because of loss of employment, 0 = otherwise.
Social norm preparedness	Most people who are important to me would think that I ought to take actions to prevent becoming infected by the coronavirus, like social distancing and regular handwashing. 1 = strongly disagree, to 7 = strongly agree.
Below the threshold of concern	Please tell me if you strongly agree, agree, neither agree nor disagree, disagree or strongly disagree with the following: The probability of being infected by the coronavirus is so low that I am not concerned about its consequences for my health. 1 = strongly disagree, to 7 = strongly agree.
Worry for COVID‐19	I am currently worried about the danger of becoming infected by the coronavirus. 1 = strongly disagree, to 7 = strongly agree.
Trust in the government response to COVID‐19	How large is your trust in how the Dutch government deals with the coronavirus? 0 = no trust at all, to 10 = trust completely.
Prefers present‐biased politicians	I rather vote for politicians who focus on solving short‐term problems than on politicians who focus on solving long‐term problems. 1 = strongly disagree, to 7 = strongly agree.
Prefers risk‐averse politicians	I am in favour of government spending on preventing or preparing for future risks even when this does not come with any short‐term benefits. 1 = strongly disagree, to 7 = strongly agree.
Risk seeking	Are you in general a person who is willing to take risks, or do you prefer to avoid risks? 0 = not willing to take risks at all, to 10 = very willing to take risks.
Self‐employed	1 = employment status is self‐employed, 0 = otherwise.
Age	Age of respondent in years.
Female	1 = respondent is female, 0 = respondent = male.
Education	Highest completed education, 1 = none or elementary education, to 7 = master or post‐doctoral degree.
Income	1 = less than €1,000, to 8 = €10,000 or more after tax monthly household income.

### 3.2 Variables for testing our hypotheses

Respondents faced several questions in relation to COVID‐19, particularly regarding their risk perceptions, individual prevention measures, personal experiences, behavioral motivations with respect to threshold models, worry, herding, and preferences for present‐biased and risk‐averse politicians. The selection of these questions is based on the behavioral biases identified in Botzen et al. (2021) of the ways in which individuals may react and respond to COVID‐19 risk; furthermore, such selection allows for testing our core hypotheses in Section 2.

Perceptions of the infection probability were asked according to both qualitative and quantitative measures. The quantitative question displayed the probability answer options on a logarithmic scale, which has been shown to effectively perform in terms of eliciting low likelihood risks attached to infectious diseases (de Bruin Parker et al., 2011; Woloshin et al., 2000). Moreover, two perceived consequences of COVID‐19 were obtained from respondents (i.e., health and financial consequences), both of which were asked on qualitative scales.

With respect to the prevention questions, they included common measures that respondents can undertake at the individual level to minimize the chance that they become infected by COVID‐19. These measures include the frequency with which individuals wash their hands, stay home, refrain from receiving guests in their home, keep a safe distance from other people when outside, and avoid public transport. All of these measures align with the basic recommendations provided by the Dutch government at the time the survey was implemented.

The COVID‐19 experiences that were elicited from the respondents related to whether they have been personally infected by the virus (and if yes, how sick they became), and whether they know at least one household member, close relative, or friend, who has been infected (and if yes, how many, and the question of whether at least one died as a consequence of infection was also asked), as well as the household expenses incurred as a consequence of COVID‐19. These expenses were subcategorized by loss of employment, temporary leave, and medical expenses.

For those questions concerning the respondents’ behavioral motivations of how they may respond to COVID‐19 risk, some questions were adopted according to previous literature and adapted to fit our context. Items used by Robinson and Botzen (2018; 2019) and Botzen et al. (2015) were utilized as a basis for investigating whether individuals follow threshold models when considering the likelihood of becoming infected by COVID‐19; those items were likewise used for assessing the respondents’ levels of worry about becoming infected with COVID‐19, and the economic consequences of the virus. Herding was assessed using an item that is similar to the one that has been adopted by a range of previous studies that examined subjective social norms in diverse domains of decision making (e.g., Abraham & Sheeran, 2004; Chang & Watchravesringkan, 2018; Fishbein et al., 1994; Latimer & Martin Ginis, 2005; Ojala, 2012). Moreover, the levels of preferences for politicians who exhibit present bias by focusing on solving short‐term problems, as well as the level of risk aversion by favoring spending on preventing or preparing for future risks at the possible expense of short‐term benefits, were asked. This risk aversion indicator is explicitly elicited in the context of future risks and hence includes a time dimension that is relevant for the NIMTOF bias.^5^

### 3.3 Other explanatory variables

In addition to the aforementioned variables for testing our core hypotheses, we obtained several other variables that may relate with COVID‐19 risk perceptions and prevention behavior which are included in the analysis. These variables include trust, risk preferences, local indicators of COVID‐19 risks, and socioeconomic characteristics.

Dryhurst et al. (2020) indicate that general trust in science has a small effect on concerns about COVID‐19. Siegrist et al. (2021) distinguish between general trust, general confidence, and social trust. They reveal that general trust and general confidence are negatively correlated with the perceived health consequences of COVID‐19; additionally, general trust negatively relates with the acceptance of government prevention measures, whereas this effect is positive for general confidence. Moreover, they observe a positive effect of social trust on the perceived health consequences of COVID‐19 and acceptance of government prevention measures. We measured trust in the government response to COVID‐19, which is closest to the social trust indicator of Siegrist et al. (2021).^6^ Hence, we expect that trust positively relates to how often individuals engage in prevention actions that are advised by the government and their support for government COVID‐19 prevention policies. Trust in the government response to COVID‐19 was obtained following a similar format to how general trust in people is determined in the European Social Survey (ESS) on an 11‐point scale.

The respondents’ general preferences toward risk were derived following an item used by Dohmen et al. (2011), which is validated in the sense that it is strongly correlated with the way in which individuals make choices in paid lottery experimental decisions and has been shown to be an effective all‐around predictor of a range of risky behaviors in practice.

Moreover, we included the local rates of people who tested positively for the coronavirus, as well as death and hospitalization from COVID‐19 as regressors of perceptions and behavior in relation to COVID‐19 in the subsequent analysis. These data were extracted from the National Institute for Public Health and the Environment (RIVM) website (https://www.rivm.nl/en). These indicators are based on the cumulative numbers of individuals by municipality who tested positive, were admitted to a hospital, or died, up to the date that the respondents filled out the survey, which amounted to 49,319, 11,835, and 6078, respectively for the Netherlands as a whole on the 18th of June 2020, the first day of survey data collection. These figures appeared in the Dutch media on a daily basis and were commonly used for motivating government policies to limit COVID‐19 infections. By the time the survey was held, COVID‐19 figures were already communicated at the municipality level as well. Hence it may be expected that individuals who live in areas with higher local COVID‐19 risks according to these indicators have higher perceptions of COVID‐19 risks and more frequently engage in prevention behavior, compared with individuals who live in areas with relatively low positive tests, hospitalizations, and deaths from COVID‐19.^7^ Furthermore, the indicators are corrected for population density per municipality and the average rates for the Netherlands as a whole as of each date the survey was taken. Correcting for these aspects is necessary to account for the fact that population density naturally differs across municipalities and that cumulative numbers increase over time. As the correlations between these local COVID‐19 risk indicators are >0.8, they are included separately in the regressions to prevent multicollinearity problems.

The socioeconomic characteristics we include in our analysis are age, education, income, and gender (i.e., being female). Age and female are related to COVID‐19 risk because older individuals and males tend to experience more severe health consequences from being infected by the coronavirus (Meng et al., 2020). Education and income may relate to the awareness of COVID‐19 risk and prevention measures, as research in the United States has shown that socially vulnerable population groups perceive a higher COVID‐19 risk (Wolf et al., 2020).

### 3.4 Statistical methods

The main goal of the statistical analysis is to investigate the relationship between several variables of interest and COVID‐19 risk perceptions and prevention measures according to our core hypotheses. The risk perceptions and prevention measures are latent variables measured on ordinal scales in our survey. Therefore, ordered probit models are used for the analysis. This method of analysis accounts for the ordinal nature of these variables, prevents predicted probabilities from falling outside the unit interval (Cameron & Trivedi, 2005), and makes no assumptions regarding the interval distances between answer options (Liddell & Kruschke, 2018). We do not dichotomize ordinal variables of interest because such an approach discards potentially useful data and reduces statistical power (Fitzsimons, 2008; Irwin & McClelland, 2001). We estimate three types of models of variables that may relate to risk perceptions. Model I only includes the local indicators of COVID‐19 risk and sociodemographic control variables, Model II adds the explanatory variables of experiences with the risk, and Model III adds the variables of feelings toward the risk. This approach allows us to identify whether any significant effect on risk perception of the local indicators of COVID‐19 risk (Model I), such as the local positive test rate, is driven by experiences (Model II) or feelings toward the risk (Model III) that may be correlated with local COVID‐19 risk levels.

## 4 RESULTS

In this section, we present the descriptive statistics of COVID‐19 risk perceptions and preparedness actions as well as the results of the statistical models of the factors related to these variables.

### 4.1 COVID‐19 risk perceptions

#### 4.1.1 Perceived COVID‐19 infection probability

**FIGURE 1:**
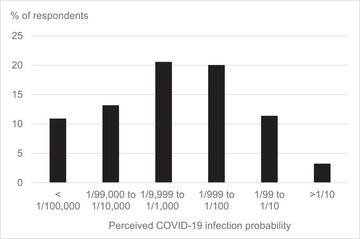
Percentage of respondents who answered a numerical category of the expected probability that they will become infected by COVID‐19 within the next year. *Note*: Up to 21% answered “do not know”

Slightly more of our respondents indicated that they are very unlikely (6%) or unlikely (21%) to become infected by COVID‐19 compared with very likely (3%) and likely (15%), whereas the majority (56%) answered the neutral option. Figure 1 illustrates that many respondents viewed becoming infected by COVID‐19 as a low probability event, as 45% of them expected this case to have a probability of 1 in 1000 or lower, whereas 35% expected that this probability is higher. At the time of our survey, it was estimated that about 5% of the Dutch population had already experienced infection by COVID‐19 (RIVM, 2020b), which suggests that most of our respondents are underestimating the probability of becoming infected themselves.

Table 2 presents the results of the ordered probit models of the qualitative perceived COVID‐19 infection probability.^8^ The results of Model I show that the perceived COVID‐19 infection probability is positively related with the local positive test rate, but this effect is not significant at the 5% level with a *p*‐value of 0.06. Of the socioeconomic variables, only age is statistically significant and is negatively related with the perceived probability, perhaps because older people are more careful and are more likely to undertake certain actions to prevent themselves from becoming infected (see findings in Section 4.2).

**TABLE 2:** Ordered probit model results of the perceived probability that they will personally become infected by COVID‐19

	Model I	Model II	Model III
	Local risk	Local risk and experience	Local risk, experience, and feelings toward risk
Local positive test rate	0.08*	0.04	0.03
Personally experienced infection	n.a.	0.31***	0.34***
Others experienced infection	n.a.	0.09***	0.08***
Below the threshold of concern	n.a.	n.a.	−0.20***
Worry for COVID‐19	n.a.	n.a.	0.21***
Age	−0.01***	−0.004***	−0.01***
Female	−0.02	0.06	0.02
Education	−0.02	−0.01	−0.03
Income	−0.02	−0.02	−0.02
Chi‐square	54.90***	327.14***	906.59***
Pseudo‐*R* ^2^	0.01	0.05	0.14
*N*	2705	2705	2705

*** *p* < 0.01.

** *p* < 0.05.

* *p* < 0.1. The dependent variable is the perceived COVID‐19 infection probability with qualitative answer options (see Table B1 of the Supporting Information for the results for the quantitative infection probability).

The results of Model II show that adding the variables about experience with COVID‐19 infections implies that the local positive test rate variable even becomes insignificant at the 10% level, suggesting that its effect in Model I is driven by the infection experience. The perceived COVID‐19 infection probability is positively related to the degree of certainty that respondents personally experienced infection by COVID‐19.^9^ The same finding holds for the degree of certainty that respondents have family members or know close relatives or friends who were infected by COVID‐19.^10^ These findings support H1a and H1b about the availability heuristic for the perceived COVID‐19 infection probability.^11^ The sizes of the coefficients suggest that personal experiences with COVID‐19 infection have a stronger relationship with risk perception than knowing others with such experiences.

Model III adds the variables of feelings toward the risk to the model, including the degree that individuals think that the COVID‐19 infection probability is too low to be concerned about and worry for COVID‐19, which are both statistically significant. The perceived COVID‐19 infection probability is positively related to worry, but it is negatively related to the threshold level of concern variable, as expected.^12^ Approximately one in four respondents at least partly agrees with the statement that the probability of becoming infected by the coronavirus is so low that she/he is not concerned about its health consequences, which indicates that the threshold model could apply to a sizable group of individuals.^13^

#### 4.1.2 Perceived health and financial consequences of COVID‐19

**FIGURE 2:**
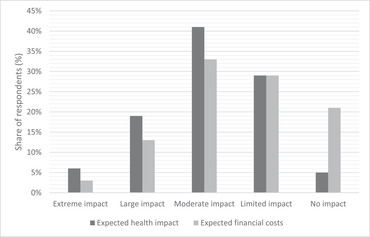
Expected health and financial consequences of COVID‐19. Note: No impact = not sick at all, no financial costs; Limited impact = a little bit sick, low financial costs; Moderate impact = quite sick, moderate financial costs; Large impact = Very sick, high financial costs; Extreme impact = Extremely sick, very high financial costs

The perceived consequences of becoming infected by COVID‐19 were obtained using the expected impacts on health and financial costs, for example due to medical costs or loss of income. As shown in Figure 2, the respondents mainly expected important health consequences and to a lesser extent financial costs.

Tables 3 and 4 present the statistical models of these perceived health and financial consequences, respectively. Model I in Table 3 of the perceived health consequences of becoming infected by COVID‐19 shows that a positive significant relationship exists with the local COVID‐19 death rate.^14^ Moreover, age is positively and significantly related with perceived health consequences, which is consistent with evidence that COVID‐19 has much more severe impacts on older people. The significantly higher perceived health consequences among females are in contrast to the observation that males in fact experience a more severe illness from COVID‐19 infection.

**TABLE 3:** Ordered probit model results of the perceived health consequences of becoming infected by COVID‐19

	Model I	Model II	Model III
	Local risk	Local risk and experience	Local risk, experience, and feelings toward risk
Local death rate	0.07***	0.07***	0.07***
Personally experienced sickness	n.a.	0.10***	0.10***
Experienced the death of someone close	n.a.	0.05	−0.001
Below the threshold of concern	n.a.	n.a.	−0.20***
Worry for COVID‐19	n.a.	n.a.	0.167***
Age	0.02***	0.02***	0.02***
Female	0.10**	0.12***	0.07
Education	0.01	0.01	−0.001
Income	−0.02	−0.02	−0.01
Chi‐square	283.20***	308.02***	799.49***
Pseudo‐*R* ^2^	0.04	0.04	0.11
*N*	2705	2705	2705

*** *p* < 0.01.

** *p* < 0.05.

* *p* < 0.1.

**TABLE 4:** Ordered probit model results of the perceived financial consequences of becoming infected by COVID‐19

	Model I	Model II	Model III
	Local risk	Local risk and experience	Local risk, experience, and feelings toward risk
Local positive test rate	0.01	0.03	0.03
Personally experienced costs from COVID‐19:			
Medical costs	n.a.	0.72***	0.64***
Costs from temporary leave	n.a.	0.79***	0.72***
Costs from employment loss	n.a.	0.72***	0.68***
Below the threshold of concern	n.a.	n.a.	−0.06***
Worry for COVID‐19	n.a.	n.a.	0.14***
Trust in the government response to COVID‐19	n.a.	n.a.	−0.02**
Self‐employed	0.60***	0.37***	0.43***
Age	−0.01***	−0.01***	−0.01***
Female	−0.11**	−0.07	−0.08*
Education	−0.01	−0.02	−0.02
Income	−0.12***	−0.11***	−0.10***
Chi‐square	171.97***	376.36***	532.94***
Pseudo‐*R* ^2^	0.02	0.05	0.07
*N*	2705	2705	2705

*** *p* < 0.01.

** *p* < 0.05.

* *p* < 0.1.

Model II in Table 3 shows the positive significant relationship between the experienced sickness and the perceived health consequences (supporting H1c). Moreover, Table B2 in Appendix B reports similar models with categorical variables of experienced sickness. These results in Table B2 show that experiencing COVID‐19 infection with no or little illness is negatively related with perceived health consequences compared with people who did not have the experience of being infected by COVID‐19.^15^ Positive and significant coefficients are observed for having been quite, very, or extremely sick after COVID‐19 infection, denoting that such individuals have higher perceived health consequences than people who were not infected by COVID‐19.^16^ These more detailed results in Table B2 confirm the general finding in Table 2 that the variable personally experienced sickness from COVID‐19 positively relates with the perceived health consequences of becoming infected by COVID‐19. Furthermore, Table 3 shows that although the local COVID‐19 death rate positively relates with perceived health consequences, experiencing a death from COVID‐19 by someone close^17^ does not have a significant effect (in contrast to H1d).^18^

Model III indicates that worry for COVID‐19 is positively and significantly related with the perceived health consequences. Moreover, the degree that individuals think that the COVID‐19 infection probability is too low to be concerned about significantly relates to the perceived health consequences in a negative manner (supporting H2a).^19^

The results of models of the perceived financial consequences of becoming infected by COVID‐19 shown in Table 4 indicate that these perceptions are not significantly related to the local positive test rate.^20^ The perceived financial consequences are significantly related to age, being female, and income, which all have negative coefficients. Moreover, we added the variable self‐employed because self‐employed individuals generally have weaker social safety nets in the Netherlands and may be more likely to suffer income losses from becoming ill. Consistent with this expectation, self‐employed individuals have significantly higher expected financial costs from becoming infected by COVID‐19.^21^ Model II illustrates that having experienced costs after being infected with COVID‐19 from medical expenses, temporary leave, and employment loss positively relates with the perceived financial consequences. Respectively, 3%, 7%, and 8% incurred such costs, suggesting that experienced financial costs were mainly related to income. Table B3 of the Supporting Information shows that the perceived financial consequences of becoming infected by COVID‐19 positively relate to the degree of certainty of having been personally infected and the extent of the experienced illness in a significant manner, but the experienced costs reported in Table 4 result in the best model fit. These findings support H1e about the availability heuristic. As indicated in Model III, worry for COVID‐19 is related to significantly higher perceived financial consequences, whereas the threshold of concern variable has a significant negative relationship with these perceptions (supporting H2a). The Dutch government has intervened extensively to limit the financial consequences of COVID‐19 through economic aid for companies. The degree of trust in how the government deals with the coronavirus is negatively associated with the perceived financial consequences of becoming infected by COVID‐19.

### 4.2 COVID‐19 prevention measures

#### 4.2.1 Individual prevention measures

**FIGURE 3:**
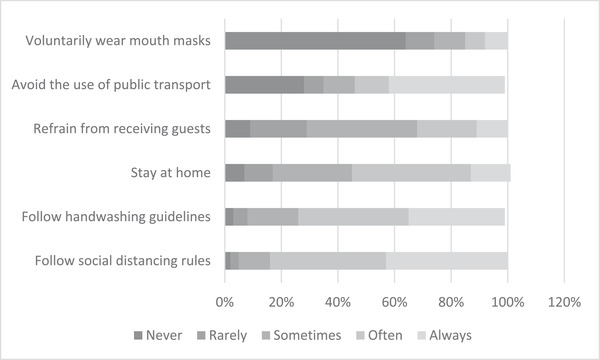
Percentage of answer options of how often respondents adopt specific measures that prevent COVID‐19 infection

Figure 3 shows how often individuals practice activities that prevent them from becoming infected by COVID‐19. The large majority follow official guidelines to regularly wash hands for at least 20 s, with 39% of the respondents adopting this practice often and 34% always. Staying at home as much as possible is also commonly practiced, with 42% of the respondents following this guideline often and 14% always. Only 11% of the respondents often refrain from receiving guests and an additional 11% always follow this guideline. Complying with social distancing rules is the most commonly practiced measure: 41% adopt this practice often and 43% always.

At the time of the survey, wearing mouth masks in public transport became compulsory in the Netherlands. Hence, to obtain an indicator of voluntary mouth mask usage, we asked the respondents about their frequency of wearing mouth masks when they do not use public transport. The majority of the respondents (64%) never take this prevention measure, whereas only 7% follow this measure often and 8% always. This result may not be surprising because at the time of the survey, the effectiveness of wearing mouth masks was actively being debated in the Netherlands. The government agency in charge of information provision on the coronavirus and prevention measures had publicly announced that the improper use of mouth masks may increase the risk of coronavirus infection instead of reducing it.

With regards to refraining from using public transport, large groups of respondents appear to be at the extremes of the answer options: 28% never follow such guidelines, whereas 41% always follow them. The reason may be that a substantial group of respondents depends on public transport for getting to work.

Table 5 presents the results of the ordered probit model of factors that relate with adopting these individual prevention measures: follow handwashing guidelines (Model A), stay at home (Model B), refrain from receiving guests (Model C), follow social distancing rules (Model D), and avoid the use public transport (Model E). Wearing mouth masks is not analyzed because its a priori relationship with risk perceptions and risk attitudes is unclear. The reason for this ambiguity is that at the time of the survey, opposite views were raised in the Netherlands about whether wearing mouth masks increases or decreases the probability of becoming infected by COVID‐19.^22^

**TABLE 5:** Ordered probit model results of the factors related with adopting these individual prevention measures: follow handwashing guidelines (model a), stay at home (model b), refrain from receiving guests (model c), follow social distancing rules (model d), and avoid the use of public transport (model e)

	Model A:	Model B:	Model C:	Model D:	Model E:
	Handwashing	Stay home	No guests	Social distancing	Avoid public transport
Local positive test rate	00.04	0.002	0.04	−0.01	0.06
Below the threshold of concern	−0.09***	−0.04***	−0.03**	−0.09***	−0.01
Perceived health consequences	0.09***	0.13***	0.04*	0.17***	0.10***
Perceived financial consequences	0.02	0.07***	0.05***	−0.06***	−0.01
Worry for COVID‐19	0.07***	0.15***	0.09***	0.03	0.06***
Trust in the government response to COVID‐19	0.06***	0.06***	0.03***	0.10***	0.04***
Social norm preparedness	0.14***	0.13***	0.08***	0.14***	0.06***
Risk seeking	−0.03**	−0.04***	−0.01	−0.05***	−0.03***
Age	0.02***	0.004**	−0.003**	0.02***	−0.01***
Female	0.32***	0.17***	0.10***	0.38***	0.10**
Education	0.01	0.05***	0.05***	0.02	0.04**
Income	−0.002	−0.02	−0.007	−0.03*	0.01
Chi‐square	684.85***	616.96***	213.64***	855.04***	151.44***
Pseudo‐*R* ^2^	0.10	0.08	0.03	0.14	0.02
*N*	2705	2705	2705	2705	2705

*** *p* < 0.01.

** *p* < 0.05.

* *p* < 0.1.

The results consistently show that the indicators of risk perceptions have a stronger relationship with individual prevention actions than the local positive test rate, which is insignificant in all models. Agreeing with the statement that the probability of being infected by the coronavirus is so low that she/he is not concerned about its health consequences negatively relates to the prevention behaviors in Table 5 (supporting H2b), except for avoiding public transport. This confirms the use of a threshold model in assessing how risk influences prevention behavior for most measures. Moreover, higher perceived health consequences are consistently related with a more regular application of the prevention behaviors (supporting H3a). Findings for the perceived financial consequences are more mixed. This variable significantly relates with more frequently complying with guidelines to stay at home and refraining from receiving guests (supporting H3a), but a negative relation is observed with how often individuals adhere to social distancing rules. The latter effect is surprising. A possible explanation is that individuals in jobs that experience high financial costs if they would be infected by COVID‐19 (e.g., self‐employed construction workers) are less able to comply with social distancing rules at work, but we cannot test this explanation with our data.

A high worry is consistently positively related with prevention behavior (supporting H3a). The degree of trust in how the government deals with the coronavirus appears to be positively associated with how frequently individuals practice the prevention behaviors that are also advised by the government. Moreover, the social norm variable is positively and significantly related with all studied prevention behaviors. Risk‐seeking individuals are less likely to engage in these behaviors, as would be expected, although this effect is insignificant for refraining to receive guests at one's home.

Of the sociodemographic variables, being female is most consistently significantly related to prevention. Females more frequently engage in all prevention actions than males. Age is also statistically significant, but the direction of its effect depends on the type of prevention action. Older individuals more frequently wash hands, stay at home, and comply with social distancing rules. However, they are less likely to refrain from receiving guests and using public transport, which may be because they are more likely to live on their own and rely more on public transport. Education level is positively related with refraining from receiving guests, staying at home, and avoiding the use of public transport, which may be because higher educated people have more possibilities to work from home.

#### 4.2.2 Support for government prevention measures

**FIGURE 4:**
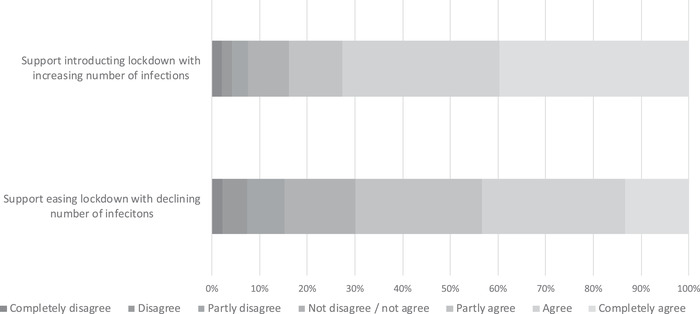
Percentage of respondents who answered the degree to which they agree with support for introducing lockdown measures when the number of COVID‐19 infections was rising, and easing these measures when the number of these infections was declining

We measured support for government prevention measures using two indicators. One of the indicators represents support for the lockdown measures when the number of coronavirus infections was rapidly increasing. The other indicator represents support for easing the lockdown measures when the number of coronavirus infections at the time of the survey was decreasing. We expected risk perceptions are positively related with the first indicator and negatively with the second indicator. Figure 4 indicates that the answers to these two questions follow a similar distribution. An exception is that substantially more respondents completely agree with introducing the lockdown measures (answered by 40%) compared with easing these measures (answered by 13%).

Table 6 shows the results of the factors related with individual support for introducing the lockdown measures for preventing COVID‐19 infections as well as the results of a separate model of support for easing these measures. The COVID‐19 risk perception variables are statistically significant and have the expected effects. The threshold of concern, perceived health consequences, and worry variables are positively related with support for the introduction of lockdown measures, and negatively related with support for easing them (supporting H2b and H3b).^23^ Moreover, trust in how the government deals with the coronavirus has a statistically significant effect on support for introducing lockdown measures that follows the same pattern as the risk perception variables.

**TABLE 6:** Ordered probit model results of the factors related with support for introducing government lockdown measures for preventing COVID‐19 infections (left column), and easing these measures (right column)

	Support introducing lockdown	Support easing lockdown
Local positive test rate	0.01	0.002
Below the threshold of concern	−0.09***	0.18 ***
Perceived health consequences	0.15***	−0.08***
Worry for COVID‐19	0.07***	−0.11 ***
Trust in the government response to COVID‐19	0.22***	−0.01
Prefers present‐biased politicians	−0.04**	0.03**
Prefers risk‐averse politicians	0.24***	0.08***
Risk seeking	−0.07***	0.001***
Age	0.01***	0.01**
Female	0.30***	0.15***
Education	0.004	0.02
Income	0.03*	0.03
Chi‐square	1250.56***	414.80***
Pseudo‐*R* ^2^	0.16	0.05
*N*	2705	2705

*** *p* < 0.01.

** *p* < 0.05.

* *p* < 0.1.

Individual preferences for present‐biased politicians are statistically significant in both models, and the pattern of results is consistent with the NIMTOF effect in adopting coronavirus prevention measures, as they negatively relate with support for the lockdown and positively with support for easing lockdown measures (supporting H5).^24^ Preferences for risk‐averse politicians have significant and positive coefficients in both models. This result is to be expected for the support of lockdown measures that aimed to limit risks when the number of infections was rapidly growing. The easing of lockdown measures increases the COVID‐19 infection risk; however, it was conducted at a moment when significant concerns were raised on the economic risks associated with prolonging the lockdown measures. The results suggest that the latter risk reduction effect was valued as important by individuals with preferences for risk‐averse politicians. Nevertheless, individuals who are personally risk seeking were less supportive of introducing the lockdown and more supportive of easing it.

## 5 DISCUSSION

### 5.1 Discussion of hypotheses

The findings underscore the importance of the availability heuristic in shaping individual perceptions of risks associated with COVID‐19. The perceived COVID‐19 infection probability is higher among respondents who personally experienced infection by COVID‐19 and who know close others who experienced COVID‐19 infection (supporting H1a and H1b). Moreover, the perceived health consequences of COVID‐19 increase with the severity of personal experience with illness from the coronavirus (supporting H1c). In this respect, a relevant aspect to note is that experiencing COVID‐19 infection with no or little illness is negatively associated with the perceived health consequences. Perhaps surprisingly, experiencing a death from COVID‐19 by someone close does not have a significant relationship with the perceived health consequences of becoming infected (not supporting H1d). Since COVID‐19 mainly causes deaths of older people with fragile health conditions, individuals may not view themselves being at higher risks if they experience such a death by someone close. Taken together, these findings suggest that the experiences of close others relate to the perceived infection probability, but not the perceived health consequences of COVID‐19; meanwhile, personal experiences are associated with both risk perception variables. Moreover, personally experiencing financial costs from COVID‐19 is positively related with the perceived financial consequences from COVID‐19 infection (supporting H1e).

Our findings suggest that the important role of the availability heuristic in shaping the perceptions of other risks, such as natural disasters (e.g., Botzen et al., 2015), is also observed for COVID‐19 risks. This finding is in line with other emerging studies that include estimates of the impacts of experience on COVID‐19 risk perceptions. Dryhurst et al. (2020) examined the influence of direct personal experience with the coronavirus on COVID‐19 risk perceptions using a survey that was implemented in 10 countries and observed a positive significant effect of experience. Other more suggestive evidence of the role of experience is provided by Meier et al. (2020). The authors revealed that individuals in March 2020 residing in Italy, where most coronavirus infections and deaths occurred in Europe, were more likely to apply prevention behavior compared with individuals in the Netherlands and Germany. This finding applied to both prevention behavior that was imposed by the government and voluntary hygienic and social measures to limit COVID‐19 infection.

The degree that individuals think that the COVID‐19 infection probability is too low to be concerned about significantly relates to the perceived health and financial consequences of COVID‐19 infection in a negative manner. This finding supports H2a and confirms that the application of this threshold model results in a simplification of an individual's assessment of risk, which has also been observed for other risks (Kunreuther, 2020; Robinson & Botzen, 2019; Slovic et al., 1977). In other words, if individuals perceive that the probability of a risk is too low to be concerned about, then they simplify the probability of the risk to zero and disregard its consequences. We observe that the application of this threshold model engenders less frequent engagement in all examined prevention actions and reduces support for government lockdown measures (supporting H2b).

Overall, we observe that perceptions of COVID‐19‐related risks have significant relationships with individual prevention behaviors (supporting H3a). In particular, the perceived health consequences are positively related to all individual prevention actions. This effect is also observed for worry for COVID‐19, except for social distancing. The latter action even has a negative relationship with the perceived financial consequences of COVID‐19 infection. The perceived financial costs do relate in the expected positive manner with staying at home and refraining from receiving guests to prevent COVID‐19 infection. Moreover, the perceived health consequences and worry variables are positively related with support for introducing lockdown measures and are negatively related with support for easing them (supporting H3b).

Our general observation that COVID‐19 risk perceptions are strongly related with demand for protection against this risk is largely consistent with other findings reported in the literature. Siegrist et al. (2021) find that the perceived COVID‐19 health risk by Swiss households increases their acceptance of government prevention measures and individual hygienic behavior; however, it is surprisingly negatively related with decreasing contact with other people. Dryhurst et al. (2020) show that individual perceptions of COVID‐19 risk are positively related with prevention behavior, including washing hands, social distancing, and wearing a mouth mask. de Bruin and Bennett (2020) conducted a survey of representative households in the United States in March; their results indicated that perceptions of COVID‐19 infection increased the likelihood of regular handwashing, but this effect was insignificant for the perceived mortality rate of COVID‐19. Furthermore, they did find that both risk perception variables were significantly and positively related with social distancing behaviors.

For all individual prevention behaviors included in our analysis, we find that individuals are more likely to apply measures that prevent infection from COVID‐19 if they believe that others think that this is the right thing to do (supporting H4). This result confirms studies that expected people to commit to behavior that prevents COVID‐19 transmission when such conduct is viewed as socially desirable by others (Soofi et al., 2020), meaning there is a social norm to do so (Van Bavel et al., 2020). This herding effect is not unique to this context, as it has been observed in prevention for other risks such as natural disasters (Lo, 2013).

Finally, we find that individual preferences for present‐biased politicians are consistent with the NIMTOF bias in adopting coronavirus prevention measures, as they negatively relate with support for the lockdown and positively with support for easing lockdown measures (supporting H5). Insofar as the NIMTOF bias is driven by voter preferences for present‐biased politicians, our finding is consistent with this bias that may contribute to a suboptimal public preparedness for pandemics. Our study has obtained the perspective of individuals by surveying households. Future research could examine the NIMTOF bias by directly studying the preferences and behaviors of politicians. Decisions about lockdown measures and their support also depend on perceived trade‐offs between the expected reduction in fatalities and the economic costs that lockdowns cause. Perceptions of these trade‐offs may depend on world views, which are found to be strong predictors for the acceptance of measures against Covid‐19 in Switzerland (Siegrist & Bearth, 2021). A limitation of our study is that we did not account for such world views.

### 5.2 Discussion of the results for other explanatory variables

We consistently find a positive relationship between worry for COVID‐19 and the perceived likelihood and consequences of COVID‐19 infection as well as with the support for government lockdown measures and individual prevention behavior, except for social distancing. Although we are unaware of other studies that explicitly focus on worry, this finding is consistent with Siegrist et al. (2021) who observe that negative associations with COVID‐19 are positively related with the perceived health consequences of COVID‐19. Siegrist et al. (2021) state that this variable of negative associations is an indicator of negative affect, and conclude that their finding confirms the importance of the affect heuristic in shaping risk perceptions (Slovic et al., 2004).

Moreover, the results of Siegrist et al. (2021) underscore that trust has complex relations with COVID‐19 risk perceptions and prevention behavior that depend on the trust indicator used. They find that social trust and general trust have opposite effects in that the former has a positive effect on the perceived health consequences of COVID‐19 and acceptance of government prevention measures, whereas general trust has a negative effect on both of these variables. Our trust variable is closest to social trust and our results indicate that it has an insignificant relationship with the perceived likelihood of COVID‐19 infection and perceived health consequences upon infection; meanwhile, the trust variable has a negative significant association with the perceived financial consequences of becoming infected by COVID‐19. Dryhurst et al. (2020) also observe a negative impact of general trust in government on COVID‐19 risk perceptions. Consistent with Siegrist et al. (2021), we observe a positive relation with trust and support for government lockdown measures; additionally, we show that this trust variable is consistently positively related with individual prevention behaviors that were advised by the government.

Our overall findings suggest that feelings toward the risk, namely trust and worry, and experiences play a larger role in shaping COVID‐19 risk perceptions and demand for protection than the local indicators of COVID‐19 risk. The local positive test rate only relates with the perceived likelihood of COVID‐19 infection. However, this effect is only significant at the 10% level, and it disappears once the effect of experience is controlled for. An exception is the local death rate that is positively related with the perceived health consequences of COVID‐19. The overall picture that emerges from our results is that feelings toward the risk and heuristics play a more important role in shaping COVID‐19 risk perception than our local indicators of COVID‐19 risk, which suggests that intuitive thinking processes dominate the way that individuals form expectations and act upon COVID‐19 risks (Kahneman, 2011).

In addition to risk perceptions, we find that individual risk attitudes have an important relationship with demand for protection against COVID‐19. In particular, individuals who are generally risk seeking are less likely to engage in individual prevention behaviors to limit COVID‐19 risk, except for refraining from receiving guests, and they are less likely to support government lockdown measures. This result supports the findings by Dohmen et al. (2011) that the employed survey question for eliciting risk attitudes explains risk reduction behaviors across a wide variety of contexts.

Moreover, with regard to sociodemographic variables, an interesting observation is that women expect more severe illness from COVID‐19 infection than men, and that women are more likely to engage in prevention behaviors; however, the perceived likelihood of becoming infected is not significantly related to gender. Older people perceive more severe health consequences, but they have lower perceptions of the likelihood that they will become infected, and the effect of age on individual prevention actions is mixed. Using simple descriptive analyses, Meier et al. (2020) also found that women in the Netherlands were more likely to apply individual prevention behavior to limit COVID‐19 infection during the early stage of the pandemic in March. The gender effect in COVID‐19 risk perceptions is also observed in international studies. Based on a survey conducted in 10 countries, Dryhurst et al. (2020) observed that COVID‐19 risk perceptions are significantly higher among females than males, but they found an insignificant effect of age. Siegrist et al. (2021) similarly found that females perceive significantly higher health risks from COVID‐19 than males, and they revealed an insignificant effect of age. Furthermore, Siegrist et al. concluded that older individuals are more likely to engage in individual prevention actions and that gender has an insignificant effect on this behavior. In reality, males and older individuals have higher health risks from COVID‐19 than females and younger individuals (Penna et al., 2020). Therefore, our findings and those from international studies suggest that males are more optimistic than women with regard to the health risks of COVID‐19.

### 5.3 Discussion of policy recommendations

Several recommendations for policies aimed at limiting pandemic risks such as COVID‐19 follow from our analysis. First, the importance of experience with infections and the availability heuristic implies that rising infections and illnesses are an opportune moment for implementing strict measures. The reason for this recommendation is that support for such measures will be high and voluntary compliance with advice to engage in risk reducing behaviors will likely be strong, such as social distancing and hygienic measures. The main challenge is how to trigger action before infections peak and when memories of experiences with infections fade. Risk communication can play a key role in triggering risk reduction behavior given the positive relationships we observe between individual perceptions of COVID‐19 risks and prevention behaviors. Individuals simplify risks and disregard risks they judge to be below a threshold level of concern. This behavior could be overcome by communication strategies that evoke the salience of the risk and encourage people to pay attention to it, for instance by stressing worst‐case scenarios (Meyer & Kunreuther, 2017). Policymakers should also take into account “pandemic‐policy fatigue,” which reduces the effectiveness of prevention policies when pandemic measures endure for a long time. This issue has been observed for COVID‐19 measures in many countries (Petherick et al., 2021; Reicher & Drury, 2021).

Another challenge is that communication strategies focused on raising risk perception should not undermine trust in the government response to COVID‐19 (Siegrist et al., 2021). Such communication strategies may use the experiences of severe health consequences in the absence of strong government and individual actions to limit infection risk. For instance, the Dutch government used the example of hospitals in London being overloaded with COVID‐19 patients to gain support for the continuation of strong lockdown measures in early 2021 to prevent a third wave of infections from the British COVID‐19 variant that was gradually spreading in the Netherlands. Such messages should be carefully designed, as Wong et al. (2021) warn against attributing responsibility for the coronavirus to foreign countries (e.g., China) in communications because this message stimulates heuristic processing of information.

Another important lesson from our study is that communication strategies should be combined with appeals to social norms, which highlight that prevention behaviors that limit the spread of the pandemic are the proper approach to adopt. Results of our study show that holding such a norm is positively related with all individual prevention actions included in our analysis. A particular challenge is how to design communication strategies that overcome the optimism observed among males with regard to the health risks of COVID‐19. Although males are in fact at higher risk, they expect lower health consequences from COVID‐19 and are less likely to engage in prevention behavior than females. Future research can focus on testing effective communication strategies for overcoming this optimism bias. In response to observations that most people tend to believe they are better off than the median person (Svenson, 1981), it was suggested by Camerer and Kunreuther (1989) to remind people what the correct statistics are to overcome this optimism bias. Males in particular have been found to have more hierarchical and individualistic worldviews than females and to be less sensitive to social inequalities resulting from risk (Finucane et al., 2000). Consequently, communication strategies that points out COVID‐19 risks at the individual level may be more effective to engage males than communication about the need for protecting vulnerable groups (e.g., the elderly), which could be investigated in future research. This argument may apply less for younger age groups for which individual risk of dying from COVID‐19 is very low, and pointing out the positive externality of COVID‐19 prevention behavior in limiting the spread of infections and related impacts on older age groups may be more effective in this case. Nevertheless, even among young people a large share experienced long‐term health complaints after COVID‐19 infection, illustrating that health risks for young age groups are not negligible.^25^

## 6 CONCLUSION

Our study adds to an emerging literature on the factors that are associated with individual perceptions of COVID‐19 risks and related decision‐making processes on prevention behaviors. Our empirical results largely support the heuristics and biases that Botzen et al. (2021) expected to be important drivers of COVID‐19 risk perceptions and behaviors based on research for other risks. In particular, our survey of 3600 households conducted in June–July 2020 in the Netherlands reveals that people simplify risk using threshold models and that risk perceptions are driven by personal experiences with COVID‐19 and experiences of close others, supporting the availability heuristic. Moreover, we observe that prevention behavior is more related to COVID‐19 risk perceptions and feelings toward the local indicators of COVID‐19 risks, and that prevention behavior is associated with herding. Support for government lockdown measures is consistent with preferences that may contribute to the NIMTOF bias.

In conclusion, our findings suggest that these behavioral biases and heuristics may be systematic features of how people perceive, and respond to, risks. Therefore, previous behavioral research can be taken as a starting point to formulate tentative expectations about behavior with regard to newly emergent risks, such as several conceptual studies did for COVID‐19 at the moment when original empirical research for this particular risk was lacking (e.g., Botzen et al., 2021; van Bavel et al., 2020). Nevertheless, empirical studies such as the current one are needed to confirm whether these expectations indeed hold, as they may not universally apply across contexts. Our results are likely to be context‐dependent, which highlights the need for future research in other countries.

## Supporting information


**Table B1**. Ordered probit model results of the perceived probability they will personally become infected by COVID‐19 with quantitative answer options
**Table B2**. Ordered probit model results of the perceived health consequences of becoming infected by COVID‐19 with categorical variables of experienced sickness (omitted baseline is not having personally been infected by COVID‐19)
**Table B3**. Ordered probit model results of the perceived financial consequences of becoming infected by COVID‐19

## Footnotes

1 We do not include the perceived COVID‐19 infection probability as risk perception indicator in the models of individual prevention behaviour since the causality is unclear. On the one hand, individuals may engage in prevention actions and support government lockdown measures because they otherwise expect the likelihood of becoming infected by COVID‐19 is too high. On the other hand, the actions and adhering to the lockdown measures lower this probability.

2 Note that we targeted homeowners as this group are typically responsible for carrying out adaptation measures against flood risk, such as the purchasing of flood insurance, which was the focus of some other questions included in the survey. We do not include any of these questions in the present article which examines COVID‐19 risk.

3 Statistics Netherlands: www.cbs.nl

4 Statistics Netherlands: www.cbs.nl

5 The variables prefer present‐biased and risk‐averse politicians have a correlation statistic of −0.15. Both can be included in the regression model since this correlation is not too high.

6 Siegrist et al. (2021) measure social trust in relation to whether individuals believe the government honestly informs the public about the COVID‐19 pandemic as well as the government's assertion that COVID‐19 has not been intentionally brought to them, and whether they believe that pharmaceutical companies act in the public interest regarding COVID‐19.

7 For instance, a high positive test rate indicates that many people at a respondent's location have been infected by COVID‐19. This indicator may signal that the coronavirus spreads rapidly at this location, which respondents may incorporate in their perceived likelihood of becoming infected by COVID‐19. This indicator of COVID‐19 risks does not capture inflection risks that people face when they travel outside of the area where they live, for example for work. However, the Dutch government strongly advised people to work from home, and most employees in the Netherlands followed this advice: namely 66% in June 2020 (RIVM, 2020a).

8 Our main analysis focusses on the perceived COVID‐19 infection probability with qualitative answer categories, instead of the similar question with quantitative answer categories since the high number (21%) of do not know responses suggests that respondents found the latter question difficult to answer. Nevertheless, we do report results of models for the question with quantitative answer categories in Table B1 in the Supporting Information with these do not know answers coded as missing values, which results in qualitatively similar findings to those reported in Table 1. Exceptions are the insignificant coefficients for the variables representing people who are convinced they have been personally infected by COVID‐19 and those who know this for certain because of test results. This insignificance may be due to the low number of people falling in these categories (4% and 2%, respectively) which makes it more difficult to detect their effect on the perceived COVID‐19 infection probability in models where the dependent variable has less observations due to missing values. An additional insight from the analyses reported in Table B1 is that high‐income individuals have lower perceptions of the quantitative infection probability.

9 Seventy‐four percent has not been personally infected by COVID‐19, 12% is uncertain about this, 7% thinks so, 4% is convinced, and for 2% this has been confirmed by test results.

10 Sixty‐seven percent does not have family members or close relatives or friends who have been infected by COVID‐19, 8% is uncertain about this, 7% thinks so, 7% is convinced, and for 11% this has been confirmed by test results.

11 One potential mechanism through which the availability heuristic works, regarding whether individuals know others who have been infected by COVID‐19, is via the size of their social network. More specifically, people who are in a larger social network, who are perhaps more likely to know individuals who have been infected by COVID‐19, are clearly also more likely to have instances or occurrences of people who have been infected that can be brought to mind.

12 Moreover, we examined whether trust in how the governments deal with the coronavirus is statistically significant in the model of the perceived COVID‐19 infection probability, which did not turn out to be the case (detailed results not reported here). All other findings are robust to the inclusion of this trust variable.

13 In particular, 8% strongly disagrees with the statement, 20% disagrees, 18% partly disagrees, 26% neither agrees nor disagrees, 15% partly agrees, 9% agrees, and 3% strongly agrees.

14 We also estimated a separate model in which the local death rate was replaced with the COVID‐19 hospitalization rate which turned out to be statistically insignificant.

15 Of the respondents who were infected by COVID‐19, 3% became extremely sick, 16% very sick, 31% quite sick, 36% a little bit sick, and 14% not sick at all.

16 Moreover, the variable “personally experienced infection” is insignificant as expected since it is not the experience of infection that is associated with perceived health consequences but experienced illness (detailed results not reported here).

17 Seven percent answered they have a family member, close relative, or close friend who died from COVID‐19. The excluded baseline category does not have a family member, close relative, or close friend who was infected or was infected but has not died from COVID‐19.

18 Moreover, the variable “others experienced infection” is insignificant as expected since it is not the experience of infection that is associated with perceived health consequences, but experienced illness (detailed results not reported here).

19 Moreover, we examined whether trust in how the government deals with the coronavirus is statistically significant in the model of the perceived COVID‐19 health consequences, which did not turn out to be the case (detailed results not reported here). All other findings are robust to the inclusion of this trust variable.

20 The local hospitalization and death rates are also statistically insignificant (detailed results not reported here).

21 The excluded baseline categories of employment status are being unemployed, student, retired, full time or part time employee, or other. A model with all employment status dummy variables and unemployed as excluded baseline category revealed that only the self‐employed variable is statistically significant (detailed results not reported here).

22 A more relevant explanatory variable for wearing mouth masks than those included in Table 5 would be whether individuals expect the measure is effective in reducing COVID‐19 infections, which was not included in our survey given our focus on risk perceptions and the hypotheses stated in Section 2.

23 The variable perceived financial consequences is not a suitable indicator of risk perceptions in these models and is hence excluded. The reason is that individuals in vulnerable jobs are likely to perceive high financial consequences from being infected by COVID‐19, which are also the kind of jobs that are most likely to suffer from the lockdown (e.g., temporary jobs in restaurants, bars, and tourism).

24 About 9%, 7%, and 2% partly agree, agree, or completely agree with the statement “I rather vote for politicians who focus on solving short‐term problems than on politicians who focus on solving long‐term problems,” while 38% answers the neutral option and 12% partly disagrees, 20% disagrees, and 14% completely disagrees with the statement. This suggests that a minority prefers present‐biased politicians, although it is a nonnegligible group.

25 The number of people under 30 years old that died from COVID‐19 in the Netherlands currently amount to 0.1% of the total fatality number, while 25% of people in the age range 16–25 who were infected by COVID‐19 experienced health complaints for longer than 3 months after COVID‐19 infection (RIVM, 2021b).
