# Supplementary material for: Behavioral biases and heuristics in perceptions of COVID‐19 risks and prevention decisions
Source: Risk Anal. 2022 Jan 29;42(12):2671–90. doi: 10.1111/risa.13882 (PMC10078638; doi:10.1111/risa.13882)
Supplement: Supplementary file 1 — Table B1. Ordered probit model results of the perceived probability they will personally become infected by COVID‐19 with quantitative answer options Table B2. Ordered probit model results of the perceived health consequences of becoming infected by COVID‐19 with categorical variables of experienced sickness (omitted baseline is not having personally been infected by COVID‐19) Table B3. Ordered probit model results of the perceived financial consequences of becoming infected by COVID‐19 [file RISA-42-2671-s001.docx]

**ONLINE SUPPORTING INFORMATION**

**Appendix A. COVID-19 Survey Questions**

The original wording of the COVID-19 questions included in the survey is given below in italic.

*The next questions will be about the current pandemic of the COVID-19 virus, which we refer to as the coronavirus.*

**Perceived likelihood (qualitative)**

*How likely do you think it is that you will personally be infected by the coronavirus?*

1. *Very likely*
2. *Likely*
3. *Not likely/not unlikely*
4. *Unlikely*
5. *Very unlikely*

**Perceived likelihood**

*Within the next year, what is your best estimate of the likelihood that you will personally be infected by the coronavirus?*

1. *Greater than 1 in 10*
2. *Between 1 in 10 and 1 in 99*
3. *Between 1 in 100 and 1 in 999*
4. *Between 1 in 1,000 and 1 in 9,999*
5. *Between 1 in 10,000 and 1 in 99,999*
6. *Less than 1 in 100,000*
7. *Not sure*

**Threshold of concern**

*Please tell me if you strongly agree, agree, neither agree nor disagree, disagree or strongly disagree with the following: The probability of being infected by the coronavirus is so low that I am not concerned about its consequences for my health.*

1. *Strongly disagree*
2. *Disagree*
3. *Partly disagree*
4. *Neither agree nor disagree*
5. *Partly agree*
6. *Agree*
7. *Strongly agree*

**Perceived health consequences**

*Suppose you would be infected by the coronavirus, how sick do you expect to get from the virus?*

1. *Extremely sick*
2. *Very sick*
3. *Quite sick*
4. *A little bit sick*
5. *Not sick at all*

**Perceived financial consequences**

*Suppose you would be infected by the coronavirus, what financial consequences do you expect for you personally from this infection, for example due to medical costs or income loss?*

1. *Very high financial costs*
2. *High financial costs*
3. *Moderate financial costs*
4. *Low financial costs*
5. *No financial costs*

*In how far do you agree with the following statements:*

**Ex ante worry pandemics**

*Before the coronavirus, I was already worried about the risk of a global pandemic.*

1. *Strongly disagree*
2. *Disagree*
3. *Partly disagree*
4. *Neither agree nor disagree*
5. *Partly agree*
6. *Agree*
7. *Strongly agree*

**Worry COVID-19**

*I am currently worried about the danger of becoming infected by the coronavirus.*

1. *Strongly disagree*
2. *Disagree*
3. *Partly disagree*
4. *Neither agree nor disagree*
5. *Partly agree*
6. *Agree*
7. *Strongly agree*

**Personal experience COVID-19 infection**

*Have you personally been infected by the corona virus?*

1. *Yes for certain because this was confirmed by test results*
2. *Yes for certain because I am convinced, but this was not confirmed by test results*
3. *I think so, but I am not fully certain about this*
4. *No*
5. *No idea*

**Personal experience COVID-19 sickness**

*How sick did you get from the (possible) infection with the coronavirus?*

1. *Extremely sick*
2. *Very sick*
3. *Quite sick*
4. *A little bit sick*
5. *Not sick at all*

**Experienced costs from COVID-19**

*Did your household incur costs as a consequence of the coronavirus for example because of the loss of employment, temporary leave, or medical expenses?*

1. *Yes, because of loss of employment*
2. *Yes, because of temporary leave*
3. *Yes, because of medical expenses*
4. *No*

**Experience COVID-19 by close others**

*Has at least one of your household members, close relatives or close friends been infected by the corona virus?*

1. *Yes for certain because this was confirmed by test results*
2. *Yes for certain because I am convinced, but this was not confirmed by test results*
3. *I think so, but I am not fully certain about this*
4. *No*
5. *No idea*

**Number of others with COVID-19 experience**

*Approximately how many* *of your household members, close relatives or close friends have been infected by the corona virus?*

1. *One*
2. *Between 2 and 5*
3. *Between 6 and 10*
4. *More than 10*

**Death others COVID-19**

*Has one of these people died as a consequence of the corona virus?*

1. *Yes*
2. *No*

**Prevention actions**

*How often do you take the following actions to prevent becoming infected by the corona virus?*

*I follow official guidelines to regularly wash hands for at least 20 seconds*

*I stay inside my house as much as possible*

*I refrain from receiving guests in my home*

*I follow official guidelines to keep distance from other people when I go outside*

*I wear a mouth mask when I am outside and do not use public transport*

*I do not use public transport because of the coronavirus*

1. *Never*
2. *Rarely*
3. *Sometimes*
4. *Regularly*
5. *Always*

*In how far do you agree with the following statements:*

**Support public prevention**

*I supported the lockdown measures when the number of coronavirus infections was quickly rising.*

1. *Strongly disagree*
2. *Disagree*
3. *Partly disagree*
4. *Neither agree nor disagree*
5. *Partly agree*
6. *Agree*
7. *Strongly agree*

**Support easing public prevention**

*I am in favour of easing the lockdown measures now the number of coronavirus infections decreases.*

1. *Strongly disagree*
2. *Disagree*
3. *Partly disagree*
4. *Neither agree nor disagree*
5. *Partly agree*
6. *Agree*
7. *Strongly agree*

**Social norm**

*Most people who are important to me would think that I ought to take actions to prevent becoming infected by the coronavirus, like social distancing and regular handwashing.*

1. *Strongly disagree*
2. *Disagree*
3. *Partly disagree*
4. *Neither agree nor disagree*
5. *Partly agree*
6. *Agree*
7. *Strongly agree*

*In how far do you agree with the following statements:*

**Preference present-biased politicians**

*I rather vote for politicians who focus on solving short-term problems than on politicians who focus on solving long-term problems*

1. *Strongly disagree*
2. *Disagree*
3. *Partly disagree*
4. *Neither agree nor disagree*
5. *Partly agree*
6. *Agree*
7. *Strongly agree*

**Preference for risk averse politicians**

*I am in favour of government spending on preventing or preparing for future risks even when this does not come with any short-term benefits*

1. *Strongly disagree*
2. *Disagree*
3. *Partly disagree*
4. *Neither agree nor disagree*
5. *Partly agree*
6. *Agree*
7. *Strongly agree*

**Trust in the government**

*How large is your trust in how the Dutch government deals with the coronavirus? Please give a grade on a scale from 0 to 10, where 0 means ‘no trust at all’ and 10 means ‘trust completely’.*

**Risk attitudes**

*Are you in general a person who is willing to take risks, or do you prefer to avoid risks?*

*Use a scale from 0 to 10, where 0 stands for not willing to take risks at all, and 10 stands for very willing to take risks….*

**Appendix B. Supplementary Results**

**Table B1.** Ordered Probit Model Results of the Perceived Probability They Will Personally Become Infected by COVID-19 with Quantitative Answer Options

|  | **Model I** | **Model II** | **Model III** |
| --- | --- | --- | --- |
|  | **Expert risk** | **Expert risk and experience** | **Expert risk, experience, and feelings toward risk** |
| Local positive test rate | .04 | .02 | .01 |
| Personally experienced infection | *n.a.* | .06** | .07*** |
| Others experienced infection: | *n.a.* | .08*** | .07*** |
| Below the threshold of concern | *n.a.* | *n.a.* | -.23*** |
| Worry for COVID-19 | *n.a.* | *n.a.* | .09*** |
| Age | -.01*** | -.01*** | -.01*** |
| Female | .08** | .10** | .05 |
| Education | .02 | .03* | .02 |
| Income | -.05*** | -.05*** | -.05*** |
| Chi-square | 107.53*** | 96.01*** | 475.04*** |
| Psuedo-R2 | 0.01 | 0.01 | 0.06 |
| N | 2,256 | 2,256 | 2,256 |

Notes: ^***^p < 0.01; ^**^p < 0.05; ^*^p < 0.1. The dependent variable is the Perceived COVID-19 infection probability with quantitative answer options.

Table B2 shows the relationship between the experienced sickness and the perceived health consequences with categorical variables of experienced sickness. Compared with the excluded baseline category of not having been infected by COVID-19, perceived health consequences from COVID-19 are significantly lower among individuals who were infected and did not get sick at all or only a little bit sick. This result implies that experiencing COVID-19 infection with no or little illness reduces perceived health consequences compared with people who did not have the experience of being infected by COVID-19. Positive and significant coefficients are observed for having been quite, very, or extremely sick after COVID-19 infection, denoting that such individuals have higher perceived health consequences (supporting H1c).

**Table B2.** Ordered Probit Model Results of the Perceived Health Consequences of Becoming Infected by COVID-19 with Categorical Variables of Experienced Sickness (Omitted Baseline Is Not Having Personally Been Infected by COVID-19)

|  | **Model I** | **Model II** | **Model III** |
| --- | --- | --- | --- |
|  | **Local risk** | **Local risk and experience** | **Local risk, experience, and feelings towards risk** |
| Local death rate | .07*** | .07*** | .06*** |
| Personally experienced sickness: |  |  |  |
| Not sick after infection | *n.a.* | -.83*** | -.92*** |
| A little bit sick | *n.a.* | -.22** | -.26*** |
| Quite sick | *n.a.* | .21** | .19* |
| Very sick | *n.a.* | .87*** | .91*** |
| Extremely sick |  | 1.14*** | 1.24*** |
| Experienced the death of someone close |  | .07 | .01 |
| Below the threshold of concern | *n.a.* | *n.a.* | -.19*** |
| Worry for COVID-19 | *n.a.* | *n.a.* | .178*** |
| Age | .02*** | .02*** | .02*** |
| Female | .10** | .09** | .05 |
| Education | .01 | .01 | -.002 |
| Income | -.02 | -.02 | -.01 |
| Chi-square | 283.20*** | 379.61*** | 883.91*** |
| Psuedo-R^2^ | 0.04 | 0.05 | 0.12 |
| N | 2,705 | 2,705 | 2,705 |

Note: ^***^p < 0.01; ^**^p < 0.05; ^*^p < 0.1.

**Table B3.** Ordered Probit Model Results of the Perceived Financial Consequences of Becoming Infected by COVID-19

|  | **Model IIa** | **Model IIb** |
| --- | --- | --- |
|  | **Local risk and experience** | **Local risk and experience** |
| Local positive test rate | . .006 | .02 |
| Personally experienced infection | .16*** | *n.a.* |
| Personally experienced sickness | *n.a.* | .18*** |
| Self-employed | .63*** | . .65*** |
| Age | -.01*** | -.01*** |
| Female | -.06 | -.07* |
| Education | -.01 | -.014 |
| Income | -.12*** | -.12*** |
| Chi-square | 220.44*** | 240.95*** |
| Psuedo-R2 | 0.03 | 0.03 |
| N | 2,705 | 2,705 |

Note: ^***^p < 0.01; ^**^p < 0.05; ^*^p < 0.1.
